# Second Primary Tumors in Patients with Gastrointestinal Stromal Tumors: A Single-Center Experience

**DOI:** 10.3390/medicina57050494

**Published:** 2021-05-13

**Authors:** Murat Koçer, Sadık Muallaoğlu, Bülent Çetin, Hasan Şenol Coşkun, Nermin Karahan, Osman Gürdal

**Affiliations:** 1 Medical Oncology Subdivision, Department of Internal Medicine, Antalya Training and Research Hospital, Health Sciences University, Muratpaşa, Antalya 07100, Turkey; 2 Medical Oncology Clinic, Private Iskenderun Gelişim Hospital, Iskenderun 31200, Turkey; smuallaoglu@hotmail.com; 3 Medical Oncology Subdivision, Department of Internal Medicine, Süleyman Demirel University Faculty of Medicine, Isparta 32260, Turkey; bulentcetin@sdu.edu.tr; 4 Medical Oncology Subdivision, Department of Internal Medicine, Akdeniz University Faculty of Medicine, Konyaaltı, Antalya 07070, Turkey; hs.coskun@yahoo.com; 5 Department of Pathology, Süleyman Demirel University Faculty of Medicine, Isparta 32260, Turkey; nerminkarahan@sdu.edu.tr; 6 Department of Biostatistics and Medical Informatics, Süleyman Demirel University Faculty of Medicine, Isparta 32260, Turkey; ogurdal@sdu.edu.tr

**Keywords:** gastrointestinal stromal tumor, second primary malignant tumor, coexistence

## Abstract

*Background and Objectives*: In this study, we investigated the frequency and type of second primary malignant tumors (SPMTs) accompanying gastrointestinal stromal tumors (GISTs), patient and tumor characteristics, and follow-up and survival data. *Materials and Methods*: We included 20 patients with SPMTs from a total of 103 patients with GISTs in a single center in Turkey. At the time of GIST diagnosis, patient age, sex, presentation symptoms, localization, pathological features of the tumor, stage, recurrence risk scoring for localized disease, treatments received, time of SPMT association, follow-up times, and survival analysis were recorded for each patient. Localization, histopathology, and stage of SPMT accompanying GISTs were also recorded accordingly. *Results*: SPMT was detected in 19.4% of patients with GISTs. Of the patients, 50% were men and 50% were women. The mean age at the time of diagnosis of GIST was 63.8 ± 10.81 years (range: 39–77 years). Of the GISTs, 60% were localized in the stomach, 25% in the small intestine, and 70% were at low risk. Of the SPMTs, 60% were in the gastrointestinal system. SPMTs were diagnosed as synchronous with GISTs in 50% of the patients. The mean follow-up period of the patients from the diagnosis of GIST was 45.6 (0.43–129.6) months. When the data were finalized, 5% died due to GIST, 35% died due to SPMT, and 15% died due to non-disease-related causes. *Conclusions*: SPMT was detected in 19.4% of patients with GISTs. GISTs were frequently located in the stomach, and most of them were at low risk. The most common SPMTs were gastrointestinal system tumors, and their coexistence was found to be synchronous. Most patients died due to SPMT during follow-up.

## 1. Introduction

Gastrointestinal stromal tumors (GISTs) are the most common mesenchymal tumors of the gastrointestinal system. They are rare, constituting 1% of all sarcomas and 3–5% of gastrointestinal tumors [1], and their incidence has been reported to be 10–15 per million in most studies [2]. They frequently originate from the stomach (60–70%) and small intestine (25–35%), and less frequently from the colon, rectum, and appendix (5% altogether), and the esophagus (2–3%). Rarely, they may be located extragastrointestinally (omentum, peritoneum, or mesentery) [3]. Mutations in the KIT (75%) or PDGFRA gene (10%) play a role in the pathogenesis of GISTs. Histologically, there are three types: spindle cell type (70%), epithelioid type (20%), and the mixed type (10%). Prognosis depends on the tumor size, mitosis rate, and localization [4]. The clinical presentation is variable. They may present with symptoms associated with the location of the tumor (such as abdominal pain, mass, gastrointestinal bleeding) or asymptomatic and incidental (for example, during surgical procedures for other diseases) in 20–30% of patients [5]. Surgery is the most effective treatment. Imatinib, a selective KIT tyrosine kinase inhibitor, and other tyrosine kinase inhibitors, are used as medical treatments. The use of imatinib as a neoadjuvant before surgery and as an adjuvant in patients at high risk after the operation and at the unresectable/metastatic stage, increases the response and survival rates [6,7,8]. Overall survival (OS) increases in GISTs treated with surgery and imatinib. Second primary tumor development may occur in patients, in addition to GISTs, with increasing life expectancy.

The frequency of tumors accompanying GISTs varies depending on the number of patients in different studies, selection criteria (including benign tumors in addition to malignant tumors in some studies), and follow-up periods. Some retrospective studies, mostly case series, were conducted on secondary tumors associated with GISTs. In recent years, the number of publications showing this relationship has increased accordingly. GISTs may coexist either synchronously or metachronously in different cancers [9]. There are publications in the literature showing that the association is mostly synchronous [9,10,11,12,13,14,15,16]. Synchronous associations often coexist with gastrointestinal cancers [11,17]. The association of GIST with secondary tumors ranges from 4.5% to 33% in different series (average, 13%) [9]. In the study conducted by Kramer et al., this rate was found to be 31.9% and 42%, respectively, in two different multicenter and single-center cohorts [18]. This rate was reported to be 21% in a retrospective monocentric analysis by Comandini et al. [19]. Although varying rates have been reported in literature, the association of GISTs with secondary tumors was reported to be 20% in a meta-analysis performed in 2019 [11,12]. Most studies have shown that GISTs are most commonly associated with gastrointestinal and genitourinary system tumors [11,12,14,18,20]. Further, gastrointestinal tumors frequently include stomach and colorectal cancers [10,11,12,14,15,18]. Among genitourinary tumors in men and women, prostate, bladder, kidney, breast, ovarian, and uterine tumors are more common [11,12,18]. In two different large-scale analyses conducted in 2019, it was reported that among SPMTs accompanying GISTs, hematological malignancies were lesser (6%) compared to gastrointestinal and genitourinary system tumors [11,12]. Lymphoma and leukemia are frequently observed in hematological tumors [12,19].

There are a limited number of studies in literature regarding the prognostic effects of SPMTs accompanying GISTs. There are publications, however, predicting shorter survival in cases of concurrent coexistence [18,19,21].

Only patients displaying second primary malignant tumor (SPMT) associations during follow-up after an initial GIST diagnosis were included in this study. We aimed to describe the frequency and type of SPMT detected in patients with GIST, the characteristics of the patients and tumors, the time of occurrence of SPMTs (pre-synchronous-post-GIST), and follow-up data for these patients. We believe that this information will accordingly contribute to the literature available on this subject.

## 2. Materials and Methods

### 2.1. Patients

We included a total of 20 patients with accompanying SPMT who were histopathologically diagnosed with GIST (*n* = 103) and admitted to the Süleyman Demirel University Medical Oncology Unit between 2002 and 2018. Patient information was retrospectively obtained from the file records. None of the patients had familial GISTs. No suspected syndromic setting was found during the follow-up.

At the time of GIST diagnosis, patient age, sex, presentation symptoms, localization, pathological features of the tumor (cell type, immunostaining for c-kit, CD-34 status, SMA, desmin, S-100, tumor diameter, mitosis, Ki-67, rupture, necrosis, and ulceration), stage according to the American Joint Committee on Cancer 8th edition TNM staging system, localization of metastases, recurrence risk scoring for localized disease (Armed Forces Institute of Pathology [AFIP] criteria, modified National Institutes of Health [NIH] Consensus Criteria), treatments received, time of SPMT association (if tumors were diagnosed simultaneously, they were defined as synchronous tumors), follow-up time, and survival analysis were recorded for each patient. In addition, localization, histopathology, and TNM stage of SPMTs accompanying GISTs were also recorded for all the patients.

Ethics Committee approval was obtained from the Süleyman Demirel University Faculty of Medicine Clinical Research Ethics Committee (13 December 2018, Study number: 256).

Patient status information was last updated in June 2020.

### 2.2. Statistical Analysis

Data are presented as mean ± standard deviation (SD). To designate and compare the characteristics of patients who were diagnosed with two different tumors, that is GIST and a second primary malignant tumor, the chi-squared test of independence and Fisher’s exact test were performed accordingly. The time from the first diagnosis to the relapse of a given event—in this study, a survival analysis of a little over 6 years was determined using Kaplan-Meier analysis. For the curve comparison, the Mantel-Haenszel log-rank test was used, which weighed all the events equally and compared the difference between the observed and expected values at each time of an event. All statistical analyses were performed using IBM SPSS version 25.0.0.2 (Statistical Package for Social Sciences in IBM SPSS Statistics for Windows, Armonk, NY, USA) and R Statistical Software v. 4.0.3 (R Foundation for Statistical Computing, Vienna, Austria). Statistical significance was defined as a two-sided α value of 0.05.

## 3. Results

### 3.1. Patients

Among the 103 GIST patients with follow-up, 20 patients with accompanying SPMT were included in the study. The incidence of SPMT in patients with GISTs was 19.4%. Of the total 20 patients, 50% (*n* = 10) were men and 50% (*n* = 10) were women. The mean age of the patients at the time of diagnosis of GIST was 63.8 ± 10.81 (39–77) years. The mean age for men was 68.3 (54–77) years and 59.3 (39–75) years for women.

### 3.2. Features of GISTs

#### 3.2.1. Symptomatology

Of the patients, 65% (*n* = 13) were asymptomatic or incidental (10 with SPMT at the same time, two during routine examinations, and one in laparotomy performed during the recurrence of SPMT), 10% (*n* = 2) had emergency operations (one obstruction and one perforation), 10% (*n* = 2) had dyspeptic complaints (heartburn, nausea, vomiting, and bloating), 5% (*n* = 1) had abdominal pain, 5% (*n* = 1) had abdominal mass, and 5% (*n* = 1) presented with complaints of abdominal pain and abdominal mass (Table 1).

#### 3.2.2. Pathological Tumor Features

Overall, 75% (*n* = 15) of the GISTs were histologically spindle cell type, 50% (*n* = 10) had tumor diameter ≤ 2 cm, and 85% (*n* = 17) had mitotic rates ≤ 5/50 HPF (*n* = 17). The mean tumor diameter was 4.63 ± 5.88 cm, and the median diameter was 2 cm (0.40–19.4 cm). Molecular analysis of the tumors was not performed in this study.

**Figure 1:**
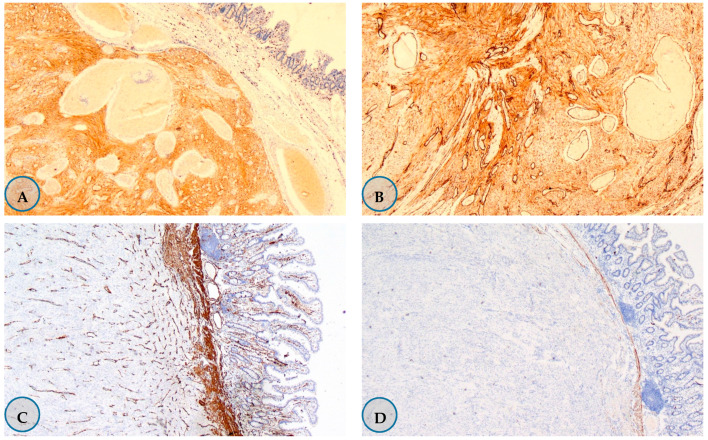

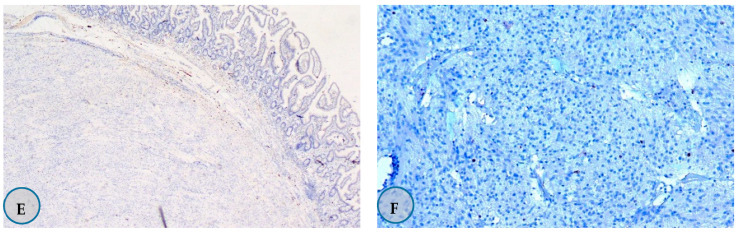
Immunohistochemistry staining samples in Gastrointestinal stromal tumor. (**A**) 85% positive staining with the C Kit ×40. (**B**) CD-34 showed more than 50% positive staining for ×200. (**C**)-SMA-negative staining in tumor, positive staining in vascular walls and muscularis mucosa ×40. (**D**) Positive staining with desmin ×40. (**E**)-Negative staining with S-100 ×40. (**F**) 2% staining with Ki-67 ×200.

Upon examination of the immunohistochemical staining features, 100% of the tumors were found to be C-kit-positive (*n* = 20), 80% were CD-34 positive (*n* = 16), 15% were SMA-positive (*n* = 3), 10% were desmin-positive (*n* = 2), and none were S-100-positive (*n* = 0) (Figure 1).

#### 3.2.3. Localization of the Tumors

The tumors were gastroesophageal in 5% (*n* = 1), located in the stomach for 60% (*n* = 12), located in the small intestine in 25% (*n* = 5), extragastrointestinal in 5% (*n* = 1), and located in the stomach and small intestine in 5% (*n* = 1) of patients. The extragastrointestinal tumor was localized to the mesentery.

#### 3.2.4. Tumor Stage-Recurrence Risk

**Figure 2:**
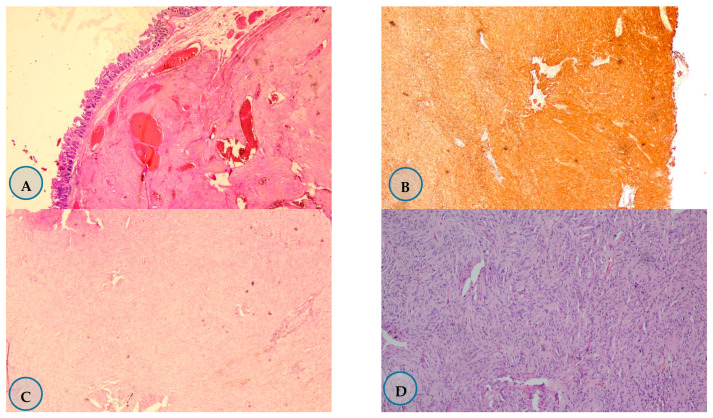
Staining samples of Gastrointestinal stromal tumors (GIST) at different stages. (**A**) Stage I jejunum localized the GIST. Hematoxylin-eosin staining of ×20. (**B**) Stage 2 Stomach-localized GIST. C Kit 100% positive staining by immunohistochemistry ×40. (**C**) Stage 3 small intestine localized GIST. Hematoxylin-eosin staining of ×40. (**D**) Stage 4 GIST with mesenteric localization. Hematoxylin-eosin staining (×200).

Patients were staged according to the American Joint Committee on Cancer 8th edition TNM staging system at the time of diagnosis. Accordingly, 70% of the patients were found to be in stage I (*n* = 14), 10% were in stage 2 (*n* = 2), 15% were in stage 3 (*n* = 3), and 5% were in stage 4 (*n* = 1). The tumor in stage 4 was located in the extragastrointestinal tract and the patient had liver metastasis (Figure 2).

Recurrence risk in non-metastatic patients (*n* = 19) was assessed according to the AFIP and modified NIH scales. In both scoring systems, 70% (*n* = 14) of the patients were found to be in the low-risk category. The pathological features of GISTs are listed in Table 2.

#### 3.2.5. Treatment

Surgery was performed in 18 out of 19 non-metastatic patients at the time of diagnosis. Of the surgeries, six were excisions, one was a wedge resection, one was a subtotal gastrectomy, four were total gastrectomy, five were segmental small bowel resections, and one was a total gastrectomy and segmented small intestine resection. In three of the five patients who underwent total gastrectomy, GISTs were removed during oncologic surgery due to gastric cancer. R0 resection was achieved in all the patients who underwent surgery. Neoadjuvant therapy was administered to one nonmetastatic patient (Case 17); however, the patient died of non-tumor-related reasons before the operation.

Only imatinib was used in the medical treatment of the patients (*n* = 3, Cases 3, 6, and 17). GIST did not progress because of imatinib hence, no other tyrosine kinase inhibitor was administered

None of the patients received adjuvant therapy. Neoadjuvant imatinib (400 mg/day) was administered to one of the three high-risk patients (case 17) according to AFIP. The patient received treatment for 14 months and died from non-tumor-related causes during follow-up before surgery. The other two patients with high risk did not receive adjuvant therapy recommended after surgery (Case 11 refused the treatment and Case 13 could not tolerate the initiated imatinib at 400 mg/day). At the time of diagnosis, one patient (Case 19) was in the metastatic stage and died on account of GIST without receiving any treatment during follow-up. Progression due to GIST was detected in only two of the 19 non-metastatic patients during follow-up (Cases 3 and 6). Both of these patients were initiated on imatinib (400 mg/day).

### 3.3. SPMT Features

The three most common SPMTs were colorectal carcinoma (*n* = 7, 35%), gastric cancer (*n* = 4, 25%), and breast carcinoma (*n* = 3, 15%). Histologically, 95% (*n* = 19) of SPMTs were epithelial tumors, and one was a hematological malignancy (multiple myeloma). Most epithelial tumors were histologically identified as adenocarcinomas. None of the GIST patients developed additional malignancies during the follow-up period. For more details about SMPT please see Table 3.

Surgery was performed on non-metastatic patients as part of the treatment for solid organ tumors. Systemic treatment decisions after surgery were made in accordance with the current treatment guidelines. Systemic therapy was administered to the metastatic cases. Patients with gastrointestinal cancer were administered five fluorouracil-based chemotherapies accordingly.

### 3.4. GIST-SPMT Emergence Time Relationship

SPMT was diagnosed before GIST in 15% (*n* = 3), synchronous with GIST in 50% (*n* = 10), and after GIST in 35% (*n* = 7) of cases. Considering the entire GIST population diagnosed in our unit (*n* = 103), SPMT was diagnosed before GIST in 2.9% of patients, synchronous with GIST in 9.7%, and after GIST (metachronous) in 6.7% of patients. In patients diagnosed with SPMT before GIST (*n* = 3), the mean time between the diagnoses of the two tumors was 118.6 ± 133.7 months (median 47 [range: 36–273] months). In patients diagnosed with SPMT after GIST (*n* = 7), the mean time between the diagnoses of the two tumors was 50.4 ± 31.4, months and the median was 45 months (range: 7–100 months). The characteristics and survival data of GIST and SPMTs are shown in Table 3.

### 3.5. Patient Follow-Up and Survival Data

The mean follow-up period of the patients from the diagnosis of GIST was 45.6 months (0.43–129.6 months). No recurrence due to GIST was detected in 17 of 20 patients during follow-up. One of the remaining three patients was metastatic at the time of diagnosis (Case 19) and died due to GIST after rapid progression of the intra-abdominal tumor mass in the follow-up without receiving any treatment. The other two patients were non-metastatic at the time of diagnosis; progression developed during follow-up, and imatinib 400 mg/day was initiated accordingly. One patient who developed recurrence (Case 6) had progressed with liver and peritoneal metastases after 8 months of follow-up. They were still alive when the study data were finalized. The other patient (Case 3) had progressed with liver and intra-abdominal metastases after 25 months. The patient died because of a secondary malignancy while receiving imatinib treatment.

When the data were finalized, 45% (*n* = 9) of the patients were alive, while 55% (*n* = 11) of the patients had died during the follow-up period. (Table 4). Of the patients who were dead, 5% (*n* = 1) were due to GIST (OS: 0.42 months), 35% (*n* = 7) were due to SPMT (mean OS: 42.24 ± 16.38 months), and 15% (*n* = 3) had died from non-disease causes (mean OS: 57.85 ± 49.54 months).

**Figure 3:**
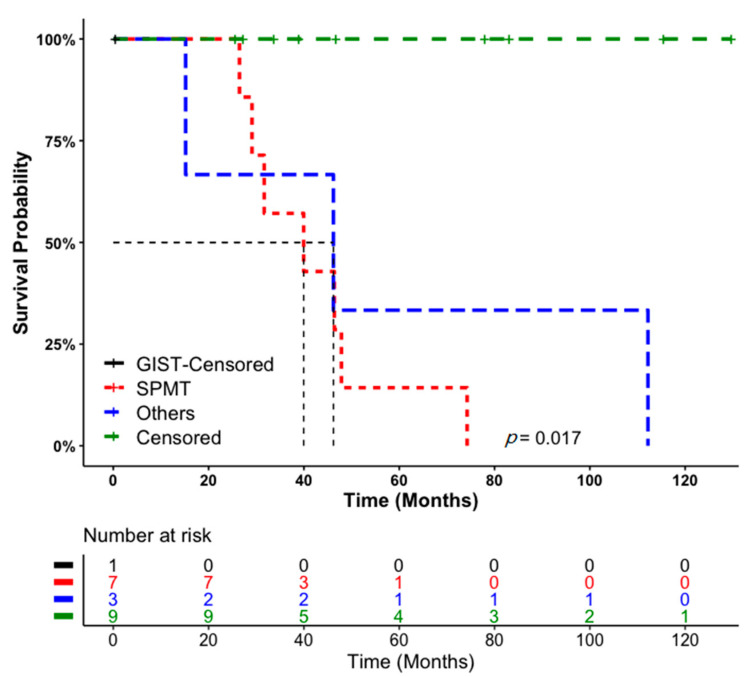
Overall survival analysis of Gastrointestinal stromal tumor (GIST). Second primary malignant tumor (SPMT), and non-tumor causes: Kaplan-Meier survival curves of survival data in relation to the causes of mortality variables, with follow-up duration of more than 6 years after first diagnoses (total *n* = 20). The numbers of patients at risk at 0, 25, 50, 75, 100, and 125 months for each of three groups are shown in the table immediately below the survival curves.

The median survival time for all patients with SPMT was 39.95 months with a confidence interval (range) of 18.70–61.19 months. When comparing GIST patients with SPMT and the others, the difference was statistically significant using the log-rank test (*p* = 0.017; Figure 3). The log-rank test indicated that there was a sharp decrease in the mortality of SPMT patients compared with the others. The Kaplan-Meier curve can be implemented to determine the median survival time with a probability of survival of 0.5. The median survival time for patients with SPMT appears to be 8.3 months versus 31 months for GIST and the others (Figure 3). 

As can also be seen from the survival curve shown in Figure 3, the proportion of patients surviving for approximately 27 months was 100%. However, the survival probability rate decreased rapidly, and after 13 months, it was approximately 42%. After 60 months, the survival probability (probability of mortality) was approximately 15%.

## 4. Discussion

Recently, there is increasing evidence available regarding the association of sporadic GISTs with secondary neoplasia [13,14,21,22,23]. In large cohorts and systematic reviews, the association between GIST and secondary malignancy has been reported to be 14–19% [24,25]. In a systematic review and meta-analysis conducted in 2019, the rate of secondary tumors with GISTs was reported to be 20% [11,12]. In our study, the association between GIST and SPMT was 19.4%, similar to the values reported in the literature.

The distribution and frequency of secondary tumors associated with GISTs may differ. Although they vary in different series in the literature, gastrointestinal and genitourinary cancers are the most common together with GISTs [1,11,12,14,18,20,23]. In our study, gastrointestinal system cancers (60%), followed by breast cancers (15%), were found to be the most common SPMTs accompanying GISTs. Secondary tumors included 35% colorectal carcinoma (25%), stomach cancer, and breast cancer (15%). Less frequent were lung, thyroid, ovarian, skin carcinoma, and multiple myeloma (the incidence of each was 5%). We believe that non-gastrointestinal tumor distributions might differ from the results of our study due to the number of patients and the heterogeneity of the studies. We did not find an association between more than one tumor in any of the GIST patients in our study. As reported in the current literature, more than one tumor can accompany GISTs, and this rate can vary between 5.3% and 20% according to different sources [9,14,15,18,23].

GIST tumor characteristics have been investigated in various studies to obtain more information on GISTs associated with secondary tumors. As mentioned in most studies, GISTs associated with secondary tumors are predominantly located in the stomach [9,13,15,18,21,23,26]; although tumor diameters vary, they are mostly below 5 cm [9,15,16,26,27]. In terms of the rates of mitosis, some studies found no mitosis, if found, the rate was mostly ≤5/50 HPF [9,15,18,23,27], although one study found a rate of >5/50 HPF [25]. In most studies, GISTs associated with secondary tumors had a low risk score [9,11,15,20,21,23,24,28]. Our results of low risk, stomach localization, small tumor diameter, and low mitosis rates are in line with other findings reported in the literature. 

Secondary tumors accompanying GISTs may occur before GIST, as well as synchronous with GIST or in follow-ups after GIST. The frequencies appear to differ though, synchronous associations have frequently been observed [9,13,14,15,16]. In a meta-analysis in which 19,627 GIST patients were evaluated, secondary tumors were found to be synchronous in 14%, metachronous in 3%, and pre-GIST in 4.6% [12]. In the evaluation of 22 studies involving 12,050 GIST patients, 50% of the second neoplasms accompanying GIST (*n* = 2426) occurred concurrently with GIST, 26% occurred before GIST, and 24% were diagnosed after GIST [11]. Some studies have shown that the frequency of SPMT is higher before GIST [22,25,26]. In our study, SPMT was diagnosed before GIST in 15% of the patients, it was synchronous with GIST in 50%, and was diagnosed after GIST diagnosis in 35% of our cases. Hence, in our study, SPMT was more common after GIST diagnosis. This frequently encountered synchronous coexistence is similar to that reported in the literature.

Secondary tumors diagnosed synchronously with GIST frequently cause intra-abdominal malignancies, though with varied incidences [15,16,21,27,29]. Diamantis et al. found synchronous intra-abdominal malignancies in 18% of the patients during the evaluation of 1108 patients with GIST. Gastric adenocarcinoma is the most common malignancy associated with GIST [29]. Waidhauser et al. determined that GISTs are most frequently synchronously observed in intra-abdominal malignancies. Of the second neoplasms, 77% occur in the gastrointestinal tract and 7% occur in the male and female urogenital tracts [11]. Out of the total 20 patients in our study, 50% were of the synchronous type. Gastrointestinal tumors constituted 90% of the synchronous SPMTs.

When GISTs are associated with secondary tumors, the time intervals for the emergence of both tumors vary. This reveals the importance of meticulous follow-up of patients, especially after GIST diagnosis. In our study, the mean time between the two tumors in patients diagnosed with SPMT before GIST diagnosis (*n* = 3) was 118.6 ± 133.7 months, and the median was 47 months (36–273 months). The mean time between two tumors in patients diagnosed with SPMT after GIST (*n* = 7) was 50.4 ± 31.4 months and the median was 45 months (range: 7–100 months). In the study performed by Rodriguez et al., the median time interval between GIST diagnosis and the second tumor in patients who developed metachronous tumors was 21.5 months [14]. In the study reported by Mayr et al. (*n* = 70), when GIST was diagnosed after secondary malignancy (*n* = 32), the mean time interval was 85 months (± 110 months) and the median interval was 50 months. If secondary malignancy was diagnosed after GIST (*n* = 21), the mean and median time intervals were 36 ± 29.8 and 29 months, respectively. In this study, secondary malignancies were frequently detected within 3 years in cases diagnosed with the first GIST (median 28.5 months) [23]. As reported by Hechtman et al., secondary malignancies developed from 5 months to 6 years after GIST, with a mean of 2.5 and a median of 2 years [25]. Murphy et al. reported that the median delay from initial cancer diagnosis to GIST diagnosis was 3.6 years for all patients, and the median time from GIST diagnosis to cancer diagnosis was 10 months for the entire cohort. In this study, a 44% increase in the prevalence of cancer before GIST diagnosis and a 66% increase in relative risk after GIST diagnosis was found in patients. The maximum increase occurred in the first year before and after GIST diagnosis [22]. Considering the number of patients and follow-up periods in our study and those reported in the literature, differences existed between the timing of the two tumor diagnoses. As the time interval for SPMT development after GIST may be short, patients should be followed carefully in this regard, and screening tests should also not be ignored accordingly.

Some studies have shown that patients with GISTs have shorter survival times than those with isolated GISTs when they are associated with secondary tumors [18,21]. In the evaluation by Kramer et al., the 5-year OS was 62.8% and 83.4% in GIST patients with and without a second tumor, respectively, and the difference was significant. There was no difference between the two groups in terms of disease-specific survival (90.8% vs. 90.9%). Median survival was 7.4 years (range: 5.9–8.8 years) and 14.8 years (range: 10.3–19.3) in GISTs with and without a second tumor, respectively [18]. In the study reported by Duo et al., GISTs without synchronous gastrointestinal malignancies had a significantly higher 5-year OS rate than those with synchronous disease (70.8% vs. 34.1%, *p* = 0.000) [21]. In a large review by Rafael-Núñez-Martin et al., 2017, the OS was worse in patients with secondary tumors with GIST than in those without secondary tumors [1]. In the study by Vassos et al., 86 patients (37 with accompanying secondary tumors) were evaluated at the end of a 60-month follow-up period. During this period, 19% of the patients died from secondary tumors, 8% died from non-tumor-related causes, and no deaths occurred due to GIST [15]. Due to insufficient data, survival could not be analyzed in the studies evaluated in the meta-analysis conducted by Petrelli et al., 2019 [12]. In our study, 35% of our patients died due to secondary tumors, 5% due to GISTs, and 15% due to non-tumor-related causes. When examining tumoral deaths, SPMTs appear to affect survival. When previously reported data and the results of our study are combined, we find that the coexistence of GISTs and SPMTs generally determines secondary tumor survival. Larger studies are however, required to confirm this hypothesis. 

GIST and SPMT treatments should be performed separately as soon as they are detected. In the nonmetastatic stage, surgery still remains the main treatment in both tumor groups. In both tumors, the necessity of systemic treatment after surgery should be performed in accordance with the current treatment guidelines. We believe that systemic treatments in both tumor groups can be performed together when necessary.

Genetic analysis of tumors is becoming increasingly important. Molecular analyses were not performed in either GISTs or SPMTs in our study. Molecular analysis of both tumor groups may be important in terms of revealing a possible common pathogenetic path between them or guiding the treatments to be applied. In this respect, large-scale studies in these patient groups may have responded to such analyses.

The limitations of our study are its retrospective and single-centered nature, lack of mutations in GISTs, and the limited number of patients.

## 5. Conclusions

We found that the incidence of GIST and SPMT was 19.4%. GISTs are frequently located in the stomach and are mostly at a low risk. They are mostly accompanied by gastrointestinal tumors; hence, GISTs are often diagnosed incidentally during gastrointestinal tumor surgery. This finding suggests that SPMTs may be a factor affecting survival because of the frequent deaths due to SPMT. We believe that our results can guide researchers and clinicians in terms of adding possible vision and awareness.

## Figures and Tables

**Table 1:** Characteristics of patients with gastrointestinal stromal tumor.

	*n* = 20 (%)
Age	
Mean Age ± SD	63.8 ± 10.81
Median age (years)	68 (39–77)
Sex	
Male	10 (50)
Female	10 (50)
Symptomatology	
Asymptomatic	13 (65)
Obstruction of small bowel	1 (5)
Perforation of the small bowel	1 (5)
Abdominal pain	1 (5)
Mass in the abdomen	1 (5)
Abdominal pain + abdominal mass	1 (5)
Dyspeptic complaints (heartburn, nausea, vomiting, bloating)	2 (10)
Treatment	
Yes	19 (95)
Surgery (R0/R1)	18 (90) (100/0)
Neoadjuvant therapy	1 (5)
Adjuvant therapy	0 (0)
No	1 (5)

SD = Standard deviation.

**Table 2:** Pathological features of gastrointestinal stromal tumors.

Tumor Characteristics		*n* = 20 (%)
Tm diameter (cm)	≤2	10 (50)
	>2, ≤5	5 (25)
	>5, ≤10	1 (5)
	>10	4 (20)
Mitosis rate (HPF)	≤5/50	17 (85)
	>5/50	3 (15)
Ki-67 (Unknown/0–9/≥10)		3/15/2 (15/75/10)
Rupture (Yes/No/Unknown)		0/20/0 (0/100/0)
Necrosis (Yes/No/Unknown)		3/11/6 (15/55/30)
Ulceration (Yes/No/Unknown)		2/10/8 (10/50/40)
Cell type (Spindle/Epithelioid/Mixt (Spindle + Epithelioid)/Unknown)		15/0/1/4 (75/0/5/20)
Immunohistochemical Staining Properties	C Kit (Positive/Negative/Unknown)	20/0/0 (100/0/0)
	CD 34 (Positive/Negative/Unknown)	16/2/2 (80/10/10)
	SMA (Positive/Negative/Unknown)	3/12/5 (15/60/25)
	Desmin (Positive/Negative/Unknown)	2/9/9 (10/45/45)
	S-100 (Positive/Negative/Unknown)	0/13/7 (0/65/35)
Recurrence risk (*n* = 19)	AFIP (None/Very low/Low/Medium/High)	10/2/2/2/3 (50/10/10/10/15)
	Modified NIH (Very low/Low/Mid/High)	10/4/0/5 (50/20/0/25)
Localization	Esophagogastric	1 (5)
	Stomach	12 (60)
	Small intestine	5 (25)
	Small intestine + Stomach	1 (5)
	Extragastrointestinal	1 (5)
Stage (TNM)	1	14 (70)
	2	2 (10)
	3	3 (15)
	4	1 (5)

AFIP, Armed Forces Institute of Pathology; NIH, National Institutes of Health; TNM, tumor-node-metastasis.

**Table 3:** Characteristics and survival data of GIST and SPMTs.

Patients				Gastrointestinal Stromal Tumor					Second Primary Malign Tumor				
No.	M/F	Age (y)	Localization	Symptom	TD (cm)	M (HPF)	Risk		Localization	Histological Type	Time of Diagnosis (from GIST)	Stage	Last Status
							AFIP	MNIH					
1	M	77	GO	Asymptomatic	0.70	≤5/50	NO	VL	Stomach	Adenocarcinoma -TPT	Synchronous	2	SPMT-EX
2	M	70	Stomach	Asymptomatic	0.50	≤5/50	NO	VL	Stomach	Adenocarcinoma	Synchronous	3	SPMT-EX
3	F	61	Stomach	A.P + M.A	19.40	≤5/50	Middle	High	Lung	Adenocarcinoma	50 m later	3	SPMT-EX
4	M	67	S.I	A.P	2.50	≤5/50	Low	Low	Skin	SCC in situ	82 m later	0	Alive
5	F	75	Stomach	D.Complaints	5.00	≤5/50	VL	Low	Thyroid	Papillary carcinoma	273 m before	L	NT-EX
6	F	51	Stomach	Asymptomatic	3.00	≤5/50	VL	Low	Rectum	Carcinoma in situ	100 m later	0	Alive
7	F	74	Stomach + S.I	Asymptomatic	0.40	≤5/50	NO	VL	Stomach	SRCC.Carcinoma	Synchronous	3	SPMT-EX
8	M	72	Stomach	Asymptomatic	1.00	≤5/50	NO	VL	Pancreas	Adenocarcinoma	Synchronous	1	SPMT-EX
9	F	41	S.I	Asymptomatic	6.50	≤5/50	Middle	High	Breast	DCIS	40 m later	0	Alive
10	F	61	Stomach	Asymptomatic	1.50	≤5/50	NO	VL	D.C	Adenocarcinoma	Synchronous	3	Alive
11	F	71	S.I	E.O	11.00	≤5/50	High	High	Rectum	Adenocarcinoma	45 m later	4	SPMT-EX
12	M	61	Stomach	Asymptomatic	0.70	≤5/50	NO	VL	Stomach	Adenocarcinoma-SRCC	Synchronous	2	NT-EX
13	F	62	S.I	E.O	5.00	>5/50	High	High	Breast	Invasive carcinoma	29 m later	1	Alive
14	M	70	S.I	Asymptomatic	3.50	≤5/50	Low	Low	D.C	Adenocarcinoma	Synchronous	2	Alive
15	M	74	Stomach	Asymptomatic	0.50	≤5/50	NO	VL	Sigmoid	M. Adenocarcinoma	36 m before	2	Alive
16	M	69	Stomach	Asymptomatic	0.40	≤5/50	NO	VL	H.F	M. Adenocarcinoma	Synchronous	2	Alive
17	M	69	Stomach	D.Complaints	11.90	>5/50	High	High	B.M	Multiple Myeloma	7 m later	E	NT-EX
18	M	54	Stomach	Asymptomatic	0.70	≤5/50	NO	VL	Sigmoid	Adenocarcinoma	Synchronous	4	SPMT-EX
19	F	39	EG	M.A	18.00	>5/50	Met.	Met.	Breast	Invasive carcinoma	47 m before	3	GIST-EX
20	F	58	Stomach	Asymptomatic	0.50	≤5/50	NO	VL	Ovary	Serous carcinoma	Synchronous	1	Alive

GIST, gastrointestinal stromal tumor; SPMT, second primary malignant tumor. M = Male. F = Female. Age: The patient’s age at the time of GIST diagnosis. Localization: GO, gastroesophageal SI, small intestine; EG, extragastrointestinal; HF, hepatic flexura. D.C = Descending Colon. B.M = Bone marrow. Symptom: *P* = abdominal pain. M.A = mass in the abdomen D. Complaints = dyspeptic complaints: heartburn, nausea, vomiting, bloating; EO, emergency operation; TD, tumor diameter; M, mitosis risk; AFIP, Armed Forces Institute of Pathology. MNIH = Modified National Institutes of Health; VL = very low. Met. = Metastatic. Histological type: SCC, squamous cell carcinoma; DCIS, ductal carcinoma in situ; M. adenocarcinoma, mucinous adenocarcinoma; SRCC, signet ring cell carcinoma TPT = tubulopapillary type. Time of diagnosis: months (months) Stage: L = localized, E = extensive, latest status: SPMT-EX = Ex due to a second primary malignant tumor; NT-EX = Ex from non-tumor causes; GIST-EX = Expired due to gastrointestinal stromal tumor.

**Table 4:** Follow-up and survival results of the patients.

	*n* = 20 (%)
Mean follow-up time ± SD	45.6 months ± 34.1
Final situation	
Alive	9 (45)
Expired	11 (55)
Due to GIST	1 (5)
Due to SPMT	7 (35)
Non-tumor-related	3 (15)

SD, standard deviation; GIST, gastrointestinal stromal tumor; SPMT, second primary malignant tumor.

## Data Availability

The data presented in this study are available only in this manuscript.
